# Stigma towards mental illness in Portuguese students of the integrated master’s degrees in medicine, veterinary medicine and pharmaceutical sciences

**DOI:** 10.1177/00207640251353681

**Published:** 2025-07-31

**Authors:** Beatriz V de Campos, Bárbara Almeida, Laetitia Teixeira, Alice Lopes

**Affiliations:** 1 Instituto de Ciências Biomédicas Abel Salazar, Universidade do Porto, Portugal; 2 Centro Hospitalar Universitário de Santo António, Porto, Portugal

**Keywords:** Social stigma, medical students, medical schools, mental illness, pharmacy students, veterinary students

## Abstract

**Background:**

Stigma towards mental illness is a problem faced by health science professionals, acting as a barrier to providing care and seeking help.

**Aims:**

The aim of this study was to evaluate and compare social stigma among Medical, Pharmacy and Veterinary students from the same university campus and assess the impact of their respective curricula on stigma levels.

**Method:**

We performed an observational cross-sectional study, involving first and final-year students of the Integrated Master’s in Medicine, Veterinary Medicine and Pharmaceutical Sciences, from two Institutions of the University of Porto, Portugal. An online self-report questionnaire, using the preliminary Portuguese version of the Attribution Questionnaire AQ-27, was employed. Additionally, a brief sociodemographic questionnaire was administered, also inquiring about close contact with mental illness.

**Results:**

A total of 182 students were considered for analysis. In terms of comparative analysis, first-year pharmacy students exhibited a higher score in the Segregation dimension compared to first-year veterinary medicine and final-year medicine students (*p* < .001). Younger age and a lower level of education corresponded to higher mean scores in the dimensions of Avoidance (*p* = .006 and .008) and Segregation (*p* < .001 for both). However, older students exhibited a lower mean score in the Pity dimension (*p* = .009). Students who did not report any close relationship with mental health problems demonstrate a higher mean score in the Avoidance dimension (*p* = .041), whereas those who cohabited with individuals with mental health problems demonstrated a lower mean score in the Segregation dimension (*p* = .014).

**Conclusion:**

This study emphasises the importance of critically design health sciences curricula to address mental health stigma, suggesting that structured evidence-based anti-stigma interventions, particularly those fostering empathy, may be essential to improve students’ attitudes and promote more compassionate future healthcare practice.

## Introduction

The first significant literary approach to stigma is Erving Goffman’s 1963 monograph, ‘Notes on the Management of Spoiled Identity’, where stigma is described as the result of ‘an attribute that profoundly discredits its object’, involving stereotyping and the segregation of groups. Goffman posits that stigma arises from the convergence of personal attributes and social stereotypes, culminating in the categorisation of individuals with specific characteristics as ‘unacceptable’ or ‘inferior’. In other words, stigma entails attributing pejorative characteristics, such as weakness, dishonesty or dangerousness, to specific groups, including those with mental illness (Goffman, 1963).

Current evidence supports that people with mental illness is often seen as dangerous, unpredictable and aggressive (Kopera et al., 2015). Despite growing awareness, and efforts to reduce stigma, the media reinforce such stereotypes, portraying these individuals as inherently dangerous. This miseducation shades empirical evidence suggesting that people with mental health illness are more often victims than perpetrators of violence (Bhavsar & Ventriglio, 2017). Research consistently shows that individuals with mental illness are at a significantly higher risk of experiencing violence, victimisation and social exclusion compared to the general population (Bhavsar & Bhugra, 2018).

Efforts to reduce stigma attached to mental illness have increasingly focussed on specific target groups, such as students (Bannatyne et al., 2023; Gervás et al., 2022; Thornicroft et al., 2016; Yamaguchi et al., 2013), and the impact of stigma on help-seeking, care-seeking and treatment participation among people with mental illness, along with stigma conceptualisation (Corrigan, 2004; Corrigan, Druss, & Perlick, 2014; Henderson et al., 2013; Rüsch et al., 2005). Individuals with mental illness have frequently reported stigmatising attitudes from health professionals (Lauber et al., 2006; Thornicroft et al., 2007). These attitudes have negatively affect the quality of care and treatment, and hinder recovery due to negative emotions triggered by interacting with these professionals (Knaak et al., 2017). These include being spoken to condescendingly, having physical symptoms dismissed as psychiatric and receiving substandard or delayed care. Subtle forms of exclusion and invalidation, often described as microaggressions, further reinforce feelings of inferiority and alienation (Gonzales et al., 2015).

The literature highlights that clinical exposure through curricular placements promoting contact with people with mental illness and specific anti-stigma training have been effective in reducing stigma among students (Friedrich et al., 2013; Happell et al., 2015; Rüsch et al., 2005). Empathy-based education is also recognised as a promising approach, particularly among healthcare students, preparing them to respond more ethically and effectively to the needs of patients and thereby humanising mental health care (Antoine et al., 2023; Bhugra et al., 2023).

Stigma towards people with mental health problems is a pervasive phenomenon, not only among the general population but also among doctors and other health professionals (Janoušková et al., 2017). Some studies report higher stigma levels among health professionals than the general population (Hansson et al., 2013; Lauber et al., 2006; Nordt et al., 2006; Vistorte et al., 2018), while others demonstrate the opposite (Reavley et al., 2014; Vibha et al., 2008; Winkler et al., 2016). Higher levels of stigma among health professionals can be explained by the phenomenon of physician bias (Thornicroft et al., 2007), whereas more positive attitudes can be justified by a wider knowledge of mental illness and explained by the ‘contact hypothesis’ (Alexander & Link, 2003; Desforges et al., 1991).

According to this hypothesis, interpersonal contact with individuals diagnosed with mental illness is a highly effective method of promoting acceptance and behavioural change, with superior results to those achieved through the use of theoretical content alone (Corrigan et al., 2012; Eksteen et al., 2017; Friedrich et al., 2013).

Pharmacists, along with other health professionals, play a significant role in mental health care (Frick et al., 2021; Rubio-Valera et al., 2014). The accessibility of community pharmacies allows closer contact with individuals with mental illness and provision of care to those in remote areas, distant from hospital centres. In such contexts, pharmacists themselves exert a greater impact on these populations (Frick et al., 2021).

Beyond their traditional role of advising on medications, pharmacists can contribute to enhancing adherence to treatment, optimising the use of medications and potentially reducing hospitalisations (Bell et al., 2005). The literature also identifies other potential interventions for pharmacists within healthcare multidisciplinary teams (Rubio-Valera et al., 2014). However, those seeking mental health support are often unaware of the pharmacist’s vast role in this field (Black et al., 2009). Conversely, stigma surrounding mental illness is also perceived as a barrier to the implementation of effective pharmaceutical interventions (Rubio-Valera et al., 2014; Sevak et al., 2023).

Targeting medical and pharmacy students is therefore crucial when attempting to influence attitudes towards mental illness, as they represent future professionals who will shape healthcare culture.

With regard to veterinary students, evidence of social stigma is limited. However, studies report elevated levels of self-stigma in this group, sometimes higher than among other student populations (Lokhee & Hogg, 2021; Yang et al., 2019). Furthermore, similar to practising veterinary, they experience high rates of mental health issues, including depression, anxiety and burnout, with a potential high risk of suicide (da Silva et al., 2023; Karaffa & Hancock, 2019). Corrigan differentiates between social stigma and self-stigma, but also notes that the two amplify each other. This is because self-stigma often results from internalising social stigma, forming a symbiotic relationship that discourages seeking help (Corrigan, 2004; Karaffa & Hancock, 2019). Social stigma is, in fact, a predictor of self-stigma, which ultimately influences help-seeking intentions (El-Hachem et al., 2023).

This study aims to analyse and compare levels of stigma towards mental illness among students enrolled in the Integrated Master’s in Medicine and Veterinary Medicine at the Abel Salazar Institute of Biomedical Sciences (ICBAS) and the Integrated Master’s in Pharmaceutical Sciences at the Faculty of Pharmacy of the University of Porto (FFUP), University of Porto. By including both first- and final-year students, who undergo a final year of professional training (fifth year in Pharmaceutical Sciences; sixth year in Medicine and Veterinary Medicine), this study seeks to assess whether knowledge acquisition and clinical contact in their respective academic programmes affect stigma. We hypothesise that greater exposure to mental health content, patient contact and empathy-based education may be associated with lower levels of stigma.

## Materials and methods

### Study design, participants and procedure

A cross-sectional study was conducted among first- and final-year students enrolled in the Integrated Master’s in Medicine and Veterinary Medicine at ICBAS, and the Integrated Master’s in Pharmaceutical Sciences at FFUP, University of Porto. All from the same university campus.

Data were collected via a digital questionnaire disseminated through the university’s institutional email system. Additional outreach was conducted through student associations of each programme, with representatives from the first and final years encouraged to promote participation by sharing the email invitation.

The questionnaire remained available for one month, after ethical approval from the ICBAS Ethics Committee. Participation was entirely voluntary, with no incentives or compensations offered. All data were collected anonymised.

### Instruments

Data were collected using The 27-item Attribution Questionnaire (AQ-27; Sousa et al., 2008; a preliminary version in Portuguese approved for use by the original author), along with a brief sociodemographic questionnaire, including age, gender, place of birth, level of education, nationality and marital status. Participants were also queried about close contact with individuals with mental illness, including whether they currently lived with them, and whether they had ever required professional mental health support themselves. Respondents were instructed to consider any situation of varying severity, affecting thought, mood and/or behaviour, with a significant functional or social impact, as a mental health problem. This included anxiety disorders, depression, eating disorders, bipolar disorder, schizophrenia, psychosis, personality disorders and other conditions regardless of formal medical diagnosis.

The AQ-27 features a vignette describing a patient with schizophrenia, followed by 27 statements rated on a 9-point Likert scale, from 1 (‘no or nothing’) to 9 (‘very much or completely’’), assessing nine dimensions of stigma: Responsibility, Pity, Anger, Dangerousness, Help, Segregation, Avoidance, Fear and Coercion. Some of these dimensions are correlated with discriminatory attitudes, such as Responsibility, Dangerousness, Fear, Anger, Coercion, Segregation and Avoidance. Others are correlated with attitudes of closeness and assistance, such as Help and Pity (Corrigan et al., 2003). Dimension scores are calculated by averaging the items that correspond to each dimension. Questions in the Avoidance dimension are reverse scored. The AQ-27 contains alternative vignettes, with different characteristics of the mental illness assessed, especially regarding severity. The following vignette (Portuguese version) was used in this study: ‘Harry is a 30-year-old single man with schizophrenia. Sometimes he hears voices and becomes upset. He lives alone in an apartment and works as a clerk at a large law firm. He has been hospitalised six times because of his illness’.

### Statistical analysis

Descriptive and inferential statistics were used to analyse the data. The data are presented employing measures of central tendency and dispersion for continuous variables and absolute and relative frequencies for categorical variables. To analyse the relationship between variables and between groups, different statistical tests were employed according to the type of variables involved. Analysis of variance (ANOVA) was used to compare means between three or more independent groups (e.g. age groups), and the Scheffé or Tukey tests were used for multiple comparisons. If the assumption of homogeneity of variance was not verified, multiple comparisons were performed considering the Games-Howell test. An independent samples *t*-test was employed to assess statistically significant differences between two independent groups (e.g. level of education). Pearson’s correlation coefficient was also used to quantify the linear association between the various dimensions of the AQ-27.

Analyses were conducted using SPSS Statistics, version 29.0, with a significance level of α = .05. The study considered the latest version of the Declaration of Helsinki.

## Results

### Socio-demographic characteristics

The sample comprised 182 students: 78 (42.86%) medical students, 22 (12.09%) veterinary students and 82 (45.05%) pharmacy students. Participants were grouped by age into three categories: ⩽19 years (46.15%), 20 to 24 years (43.96%) and ⩾25 years (9.89%). Most participants were female (81.32%), single (98.35%), Portuguese (99.45%) and from northern of Portugal (83.52%), with an educational level corresponding to the 12th grade (62.64%).

Regarding contact and proximity with mental health problems, 17.03% reported no personal or close contact, and 42.86% had never sought professional assistance for mental health concerns. Conversely, 25.27% reported personal contact, 23.08% had a first-degree relative and 32.97% had a relative or close friend with mental health problems. The ‘other’ category reflects overlapping experiences. Among respondents, 23.63% live with the individual in question, 40.11% have attended psychological appointments, 13.18% have seen psychiatrists and 3.30% have consulted both. ‘Other’ also includes those who sought assistance from a family physician. Full data are presented in Table 1.

**Table 1.** Socio-demographic characteristics of the sample (*n* = 182).

Variable	Frequency (*n*)	Percentage (%)
Gender		
Female	148	81.32
Male	31	17.03
Prefer not to say	3	1.65
Civil status		
Single	179	98.35
Married	2	1.10
Divorced	1	0.55
Age range		
⩽19	84	46.15
20–24	80	43.96
⩾25	18	9.89
Nationality		
Portuguese	181	99.45
Other	1	0.55
Birth place/natural from		
North	152	83.52
Centre	14	7.69
Metropolitan area of Lisbon	3	1.65
Islands	7	3.84
Foreign	3	1.65
Prefer not to say	3	1.65
Education level		
12th grade	114	62.64
University	68	37.36
Relationship with mental health problems		
Personal	46	25.27
First-degree relative	42	23.08
Close relative or friend	60	32.97
None	31	17.03
Other	3	1.65
Lives with one or more of the aforementioned individuals (with mental health problems)		
Yes	43	23.63
No	58	31.87
Not applicable	81	44.50
If ever required professional assistance due to mental health concerns, what kind of help		
Psychology appointments	73	40.11
Psychiatry appointments	24	13.18
Both	6	3.30
None	78	42.86
Other	1	0.55
Student of the Integrated Master’s Degree in		
Medicine	78	42.86
Pharmaceutical sciences	82	45.05
Veterinary medicine	22	12.09
Year and Integrated Master’s Degree		
First/Medicine	33	18.13
Sixth/Medicine	45	24.73
First/Pharmaceutical Sciences	50	27.47
Fifth/Pharmaceutical Sciences	32	17.58
First/Veterinary Medicine	16	8.79
Sixth/Veterinary Medicine	6	3.30

### AQ-27

Mean scores for the all the AQ-27 items are presented in Table 2. The Help dimension had the highest overall mean, with two items scoring above seven on average: ‘I would be willing to talk to Harry about his problems’ and ‘How likely is it that you would help Harry?’. Nevertheless, the statement with the highest average score overall was ‘If I were in charge of Harry’s treatment, I would require him to take his medication’, related to Coercion. Items related to Pity dimension also scored highly, particularly ‘How much concern would you feel for Harry?’. Followed by the Avoidance dimension, with higher mean scores for ‘If I were a landlord, I probably would rent an apartment to Harry’ and ‘If I were an employer, I would interview Harry for a job’.

**Table 2.** Descriptive analysis for each item of the AQ-27.

Item and Statement		Dimension	Min	Max	*M*	*SD*
Q1	I would feel aggravated by Harry.	Anger	1	9	3.60	1.93
Q4	How angry would you feel at Harry?		1	6	1.95	1.23
Q12	How irritated would you feel by Harry?		1	7	2.38	1.49
Q7	If I were an employer, I would interview Harry for a job.	Avoidance	1	9	6.24	2.02
Q16	I would share a car pool with Harry every day.		1	9	5.31	2.21
Q26	If I were a landlord, I probably would rent an apartment to Harry.		1	9	6.42	2.02
Q5	If I were in charge of Harry’s treatment, I would require him to take his medication.	Coercion	1	9	7.88	1.66
Q14	How much do you agree that Harry should be forced into treatment with his doctor even if he does not want to?		1	9	5.16	2.39
Q25	If I were in charge of Harry’s treatment, I would force him to live in a group home.		1	7	2.27	1.63
Q2	I would feel unsafe around Harry.	Dangerousness	1	9	3.66	1.91
Q13	How dangerous would you feel Harry is?		1	9	3.56	1.89
Q18	I would feel threatened by Harry.		1	9	2.69	1.56
Q3	Harry would terrify me.	Fear	1	9	3.57	2.00
Q19	How scared of Harry would you feel?		1	9	3.10	1.76
Q24	How frightened of Harry would you feel?		1	9	3.20	1.90
Q8	I would be willing to talk to Harry about his problems.	Help	1	9	7.79	1.56
Q20	How likely is it that you would help Harry?		1	9	7.62	1.50
Q21	How certain would you feel that you would help Harry?		2	9	6.24	1.80
Q9	I would feel pity for Harry.	Pity	1	9	6.87	2.05
Q22	How much sympathy would you feel for Harry?		1	9	6.17	2.25
Q27	How much concern would you feel for Harry?		1	9	7.35	1.51
Q10	I would think that it was Harry’s own fault that he is in the present condition.	Responsibility	1	9	1.33	0.98
Q11	How controllable, do you think, is the cause of Harry’s present condition?		1	9	4.97	2.27
Q23	How responsible, do you think, is Harry for his present condition?		1	6	1.45	0.98
Q6	I think Harry poses a risk to his neighbours unless he is hospitalised.	Segregation	1	9	3.14	1.87
Q15	I think it would be best for Harry’s community if he were put away in a psychiatric hospital.		1	9	2.88	1.80
Q17	How much do you think an asylum, where Harry can be kept away from his neighbours, is the best place for him?		1	8	2.08	1.45

*Note*. Max = maximum; Min = minimum; *SD* = standard deviation

The items with the lowest mean scores from Responsibility dimension were ‘I would think that it was Harry’s own fault that he is in the present condition’ and ‘How responsible, do you think, is Harry for his present condition?’, the latter with a maximum average score of 6. A similar score was seen for ‘How angry would you feel at Harry?’ from the Anger dimension.

Globally, the highest scoring dimensions were, in descending order, Help, Pity and Coercion. In contrast, Responsibility, Anger and Segregation scored lowest (Table 3).

**Table 3.** Descriptive analysis for each dimension in the AQ-27.

Dimension	Min	Max	*M*	*SD*
Anger	1.00	6.33	2.64	1.20
Avoidance	1.00	8.67	4.01	1.67
Coercion	1.00	8.00	5.10	1.35
Dangerousness	1.00	9.00	3.30	1.61
Fear	1.00	9.00	3.29	1.76
Help	2.67	9.00	7.21	1.30
Pity	2.00	9.00	6.80	1.48
Responsibility	1.00	6.33	2.58	1.02
Segregation	1.00	7.67	2.70	1.42

*Note*. Max = maximum; Min = minimum; *SD* = standard deviation

Table 4 shows the results of AQ-27 dimensions comparisons by degree programme and academic year. A significant difference was found in the Segregation dimension (*p* < .001), with first year Pharmacy students scoring higher in this dimension compared to first year Veterinary and the final year Medicine students. Analysis of AQ-27 items (Table A1 in Appendix) confirmed significant differences across all three items in this dimension. Notably, final-year medicine students scored significantly lower score on Q15 compared to first-year medicine students and both first- and final-year pharmacy students (*p* < .001). Additionally, Q5 (Coercion) scored significantly higher among final-year Pharmacy students in comparison to first-year medicine students and Q7 (Avoidance) scored significantly higher among first-year pharmacy students compared to final-year medicine students (*p* < .001).

**Table 4.** Comparison of AQ2-7 dimensions according to the Master’s Degree and year.

Item	*n*	*M*	SD	ANOVA (F)	*p*
Anger				0.408	.843
Avoidance				2.159	.061
Coercion				1.991	.082
Dangerousness				0.802	.550
Fear				2.119	.065
Help				0.128	.986
Pity				1.633	.154
Responsibility				0.845	.519
Segregation				8.142	<.001*
First/Medicine	33	2.75	1.32		
Sixth/Medicine	45	1.99	0.99		
First/Pharmaceutical Sciences	50	3.53	1.57		
Fifth/Pharmaceutical Sciences	32	2.80	1.21		
First/Veterinary Medicine	16	2.21	1.37		
Sixth/Veterinary Medicine	06	1.67	0.60		

*Note. SD* = standard deviation.

* *p* < .050.

Significant differences were found between integrated master’s degrees (grouping first and final-year students) in the Segregation, Coercion and Fear dimensions (Table 5). Pharmacy students exhibited a higher mean score than medical students in the Fear dimension (*p* = .007), and higher than both medical and veterinary students in Coercion (*p* = .007) and Segregation (*p* < .001) dimensions.

**Table 5.** Comparison of AQ-27 dimensions according to the Master’s Degree.

Item	*n*	*M*	*SD*	ANOVA (*F*)	*p*
Anger				0.335	.715
Avoidance				1.908	.151
Dangerousness				1.983	.141
Help				0.195	.823
Pity				2.722	.068
Responsibility				1.765	.174
Coercion				5.044	.007*
Medicine	78	4.89	1.31		
Pharmaceutical Sciences	82	5.44	1.27		
Veterinary Medicine	22	4.62	1.58		
Fear				5.097	.007*
Medicine	78	2.95	1.75		
Pharmaceutical Sciences	82	3.74	1.64		
Segregation				12.82	<.001*
Medicine	78	2.31	1.20		
Pharmaceutical Sciences	82	3.25	1.48		
Veterinary Medicine	22	2.06	1.22		

*Note. SD* = standard deviation.

* *p* < .050.

Table 6 shows significant differences in Avoidance, Pity and Segregation when comparing AQ-27 dimensions according to age range. Students aged ⩽19 years had a higher mean score than the older groups (*p* = .006 and <.001, respectively). In contrast, students aged ⩾25 years exhibited a lower mean score in Pity than the younger groups (*p* = .009).

**Table 6.** Comparison of AQ-27 dimensions according to age range.

Item	*n*	*M*	*SD*	ANOVA (*F*)	*p*
Anger				0.691	.503
Coercion				0.040	.961
Dangerousness				0.711	.493
Fear				0.527	.591
Help				0.842	.433
Responsibility				0.086	.918
Avoidance				5.183	.006*
Age ⩽19	84	4.41	1.57		
20–24	80	3.75	1.68		
⩾25	18	3.31	1.74		
Pity				4.792	.009*
Age ⩽19	84	6.92	1.35		
20–24	80	6.90	1.52		
⩾25	18	5.80	1.54		
Segregation^a^				8.033	<.001*
Age ⩽19	84	3.13	1.60		
20–24	80	2.40	1.15		
⩾25	18	2.06	0.98		

*Note. SD* = standard deviation.

* *p* < .050.

a Multiple comparison performed by the Games-Howell test

According to education level (Table 7), students with lower level of education, corresponding to the 12th grade, scored significantly higher in Avoidance and Segregation than those with higher education (university; *p* = .008 and <.001, respectively).

**Table 7.** Comparison of AQ-27 dimensions according to previous education level.

Item	*n*	*M*	*SD*	*t* (*df* = 180)	*p*
Anger				0.516	.607
Coercion				0.788	.432
Dangerousness				0.782	.435
Fear				0.866	.388
Help				0.776	.439
Pity				1.770	.078
Responsibility				0.843	.400
Avoidance				2.690	.008*
12th grade	114	4.27	1.58		
University	68	3.59	1.75		
Segregation				3.812	<.001*
12th grade	114	3.00	1.49		
University	68	2.20	1.12		

*Note. SD* = standard deviation; *df* = degrees of freedom.

* *p* < .050.

As shown in Table 8, Avoidance mean score were significantly higher among students who do not have any kind of relationship with mental health issues than among those who have a personal relationship with mental health issues and those who have a first-degree relative with a mental health problem (*p* = .041).

**Table 8.** Comparison of AQ-27 dimensions according to relationship with mental health problems.

Item	*n*	*M*	*SD*	ANOVA (*F*)	*p*
Anger				0.062	.993
Coercion				0.783	.538
Dangerousness				0.446	.775
Fear				0.095	.984
Help				0.976	.422
Pity				0.197	.939
Responsibility				0.742	.564
Segregation				2.105	.082
Avoidance				2.556	.041*
Personal	46	3.69	1.64		
First-degree relative	42	3.67	1.73		
Close relative/friend	60	4.11	1.62		
None	31	4.76	1.59		
Other	3	4.11	1.68		

*Note. SD* = standard deviation.

* *p* < .050.

Students who cohabit with individuals with mental health problems, including first-degree relatives and close family or friends, reported significantly lower mean scores in the Segregation dimension compared to those who do not (*p* = .014, Table 9).

**Table 9.** Comparison of AQ-27 dimensions according to cohabitation with individuals with mental health problems.

Item	*N*	*M*	*SD*	*t* (*df* = 99)	*p*
Anger				0	1
Avoidance				1.016	.312
Coercion				−0.796	.428
Dangerousness				0.009	.993
Fear				−0.681	.497
Help				0.325	.746
Pity				−0.390	.698
Responsibility				0.332	.74
Segregation				2.515	.014*
No cohabitation	58	2.85	1.29		
Cohabitation	43	2.19	1.31		

*Note. SD* = standard deviation; *df* = degrees of freedom.

* *p* < .050.

Finally, correlations between the nine AQ-27 dimensions are presented in Table 10.

**Table 10.** Correlation between different AQ-27 dimensions (Pearson’s correlation coefficient).

Dimension	Anger	Avoidance	Coercion	Dangerousness	Fear	Help	Pity	Responsibility	Segregation
Anger	1								
Avoidance	0.411*	1							
Coercion	0.226^a^	0.263*	1						
Dangerousness	0.674*	0.497*	0.353*	1					
Fear	0.666*	0.497*	0.362*	0.886*	1				
Help	−0.295*	−0.418*	0.036	−0.214¹	−0.188¹	1			
Pity	0.068	0.115	0.239¹	0.136	0.185¹	0.299*	1		
Responsibility	0.219¹	0.101	0.281*	0.218¹	0.208¹	−0.048	0.028	1	
Segregation	0.413*	0.477*	0.486*	0.531*	0.526*	−0.141	0.215¹	0.258*	1

a *p* < .05.

* *p* < .001.

## Discussion

This study aimed to assess differences in social stigma towards mental illness among medical, veterinary and pharmacy students. Additionally, it aimed to understand how curricula and academic progression may influence social stigma by comparing first and final-year students of their respective programmes.

Unlike previous research in Portugal that found significant differences between first- and final-year medical students, specifically in the Segregation dimension, with lower scores at the end of the course (Pinto et al., 2020), our results showed no statistically significant difference between first and final-year medical students. However, final-year students tended to achieve lower mean scores in several dimensions.

The literature remains divided on the impact of curricular plans and internships in psychiatry in medical schools. Some studies suggest that close contact with individuals with mental illnesses is an effective method of promoting acceptance and a change in attitudes, both among the general population and among medical students (Corrigan et al., 2012; Eksteen et al., 2017; Friedrich et al., 2013) and that psychiatry internships can effectively reduce stigma (Chang et al., 2017; Chiles et al., 2017; Economou et al., 2017; Janoušková et al., 2017; Sandhu et al., 2019; Shen et al., 2014; Wang et al., 2016). Others argue that in a long-term follow-up, the results are inconsistent, even indicating a decrease in the initial positive impact of the interventions (Friedrich et al., 2013; Telles-Correia et al., 2015). Consequently, some authors propose that medical education has limited impact on stigma and that even long-term contact with mentally ill patients does not necessarily result in a change in negative attitudes (Zhu et al., 2018).

Recent work suggest that the structure and quality of educational experiences may be more important than their mere inclusion in curricula. In particular, empathy-focussed training has been identified as a potential key component of successful anti-stigma interventions. Teaching empathy through simulation, reflective practices or guided patient interaction can help students to better understand the lived experience of individuals with mental illness and respond with greater compassion and ethical sensitivity. Such approaches may offer a more durable shift in attitudes by addressing emotional and interpersonal dimensions of stigma (Antoine et al., 2023; Bhugra et al., 2023). Further research is needed to better understand which pedagogical strategies most effectively support lasting attitude change.

The Integrated Master’s in Medicine (ICBAS) includes mandatory semester subjects such as medical psychology and psychiatry, incorporating reflective practices, structured patient interactions and simulation-based learning. These aim to enhance students’ understanding of both pathophysiology and psychosocial aspects of mental illness, and to reduce stigma through targeted educational strategies. While no causal relationship can be established, this may contribute to the tendency for lower stigma scores in several stigma dimensions among final-year students.

Pharmacy students exhibited statistically significant higher mean scores in Coercion, Fear and Segregation dimensions, which were found to be highly and positively correlated. This suggests a greater tendency to exclude people with mental illness from the community and also reflects the idea that students can better help these patients through medication. This aligns with findings from a Portuguese study, where a population of medical students revealed that students expressing fear and apprehension towards peers with mental illness were more likely to favour involuntary treatment in a psychiatric hospital (Moreira et al., 2021).

Although no statistically significant differences were found between first- and final-year pharmacy students, a slight downward trend was observed in Avoidance and Segregation, where the mean scores differed slightly more than in the other dimensions. Since the desire for social distancing is a way of determining stigma and the discriminatory attitude towards mental illness is correlated with higher scores in those dimensions (Cashwell et al., 2011; Corrigan et al., 2003; Link & Phelan, 2001), this may indicate a potential reduction in stigma. Still, there was a tendency for pharmacy students to score higher than medical students across all parameters, suggesting a greater overall stigma.

Pepa et al. (2021) demonstrated that a course covering topics such as the role of psychiatric pharmacists, psychotherapy, maternal mental health and new treatments, taught by leading psychiatry clinicians and designed to be highly interactive, with students being present at community psychiatric interventions, can have an impact on reducing stigma among pharmacy students (Pepa et al., 2021). In addition to theoretical knowledge, there is a growing body of literature that emphasises the significance of integrating modules and content aimed at reducing stigma surrounding mental health in pharmacy education (Aluh et al., 2022; Bamgbade et al., 2016; Sevak et al., 2023). This can even make students perceive the role of pharmacists in reducing stigma by enhancing mental health education and raising public awareness (Sevak et al., 2023).

It is important to note that the Master’s Degree in Pharmaceutical Sciences at FFUP lacks mandatory subjects focussed on understanding the pathophysiology and implications of mental health issues, as well as anti-stigma modules or interventions. The sole potential exposure to mental illness occurs during the final-year internships, typically in community pharmacies, randomly.

Since prior research suggests that direct contact with people affected by mental health problems can markedly enhance pharmacy students’ attitudes (Bamgbade et al., 2016; O’Reilly et al., 2012), these internships could potentially influence the slight improvement in stigma between first- and final-year pharmacy students. However, the limited and delayed nature of this exposure could also account for persistently high stigma levels, reinforcing that clinical exposure alone may not be sufficient to reduce stigma, particularly without structured reflection, targeted discussion or intentional anti-stigma components.

To our knowledge, this is the first study to assess social stigma among veterinary students and to compare results across students from these three academic areas. Due to considerable drop-out rates, the final-year veterinary student sample, although proportional, was too small to yield statistically meaningful comparisons with the first-year students to conclude about curricular impact in possibly changing attitudes towards mental illness. Nevertheless, when considering the broader picture, veterinary students tended to show stigma scores closer to those of medical students. Further research in this population is needed to ascertain whether targeted anti-stigma interventions, or optional curricular subjects in psychology or psychiatry, could influence stigma experienced by this population.

Comparing the three master’s degrees and the overall sample surveyed, in a manner similar to the Portuguese study conducted by Pinto et al. (2020) on a population of medical students (Pinto et al., 2020), Help, Pity and Coercion were the dimensions that scored highest, while Responsibility, Anger and Segregation scored lowest. This suggests these may be the most marked dimensions of stigma in health science students.

With regard to age, students aged ⩽19 scored higher in the dimensions of segregation and avoidance, while those aged ⩾25 exhibited lower scores in Pity. These results align with a previous study carried out on the general population of Swedish, which showed that younger age was associated with better mental health literacy and a positive intention to interact with mental illness in the future, while older age was associated with more positive attitudes in the domains of integration, avoidance and community mental health ideology (Hansson et al., 2016).

The observation that students with a lower level of education scored higher in Avoidance and Segregation, denoting greater discrimination, is consistent with the existing literature suggesting that education alone may be an effective strategy for reducing stigma by deepening understanding of the disease and the potential for treatment (Yamaguchi et al., 2011). However, the current findings do not establish a causal relationship between education alone and lower stigma levels, and this association should be interpreted with caution.

Students with no previous contact with mental health problems, either personally or through a family member or close friend, exhibited higher score in Avoidance. This is consistent with a substantial body of literature indicating that greater familiarity with individuals with mental health problems is associated with a reduction in stigma (Familiarity Effect), which is reflected in a lower desire for social distance (Aluh et al., 2022; Angermeyer et al., 2004; Corrigan, Powell, & Michaels, 2014; Eksteen et al., 2017; Griffiths et al., 2014). This is further supported by a Portuguese study in medical students, which observed a reduction in stigma among those with personal or familial contact with mental illness (Moreira et al., 2021).

Accordingly, students living with at least one individual with a mental health problem with whom they have a close relationship also exhibited lower Segregation scores, reinforcing the potential importance of close, personal contact.

Unlike Pinto et al. (2020), who found that those who had been to a psychiatric consultation had a significantly lower Segregation score, no statistically significant difference was identified in this study.

### Limitations

The main limitations of this study include its cross-sectional design and potential bias in sample selection. A more robust approach would have involved tracking the same student cohort from the first to the final year, allowing for a more accurate assessment of the impact of each educational programme on stigma.

The results are not generalisable due to the relatively small sample size, particularly among veterinary medicine students. Although proportional, this subgroup remains relatively limited.

Moreover, the results are based on self-reported attitudes, which may be subject to response bias. While the AQ-27 is a validated instrument for assessing stigma, only a preliminary version is available in Portuguese. Moreover, the existence of alternative instruments to assess social stigma in the literature limits the comparability of results.

## Conclusion

To the best of our knowledge, this was the first study to compare stigma towards mental illness across these three health disciplines and to examine the impact of their respective curricula.

The higher stigma levels observed among pharmacy students highlight the need to assess how and when anti-stigma content is introduced into the curriculum. As pharmacist’s role is increasingly recognised in community pharmacies, where they engage with patients with mental illness, it is important to consider strategies to reduce stigma throughout their training. Exploring how stigma evolves over the course of education may help identify the most effective timing and format to ensure sustained change.

Our findings emphasise the need to critically evaluate how health sciences curricula address mental health stigma. While some associations suggest that educational level and personal contact may influence attitudes, the cross-sectional nature of this study limits causal inference. It is therefore essential to avoid the assumption that education or exposure alone reduces stigma without considering the quality and context of these experiences.

Future research should explore the integration of specific evidence-based anti-stigma interventions into health education programmes. These may include structured contact-based interventions, simulation and role-play, reflective practice modules and narrative-based education using patient testimonies and lived experience. Several of these approaches centre on empathy, which may be a key to reshaping how future professionals perceive and respond to individuals experiencing mental illness.

Finally, identifying the most prominent dimensions of stigma can help guide more effective interventions. Further studies should evaluate the impact of anti-stigma educational programmes on both social and self-stigma, and their relationship with increased openness to seeking mental health care among students, as well as their potential to foster broader cultural change within healthcare.

## Appendix

**Table A1.** Comparison of AQ2-7 items according to the Master’s Degree and year.

Item	Dimension	*N*	*M*	*SD*	ANOVA (*F*)	*p*
Q1	Anger				0.607	.694
Q4					0.828	.531
Q12					0.788	.56
Q7	Avoidance				3.208	.008*
Sixth/Medicine		45	2.82	1.63		
First/Pharmaceutical Sciences		50	4.34	2.08		
Q16					1.414	.222
Q26					0.556	.734
Q5	Coercion				3.279	.007*
First/Medicine		33	7.18	1.94		
Fifth/Pharmaceutical Sciences		32	8.34	1.31		
Q14					1.155	.334
Q25					2.119	.065
Q2	Dangerousness				0.2	.962
Q13					0.95	.45
Q18					1.796	.116
Q3	Fear				2.065	.072
Q19					2.225	.054
Q24					1.658	.147
Q8	Help				0.944	.454
Q20					0.514	.766
Q21					0.119	.988
Q9	Pity				0.875	.499
Q22					1.606	.161
Q27					1.094	.365
Q10	Responsibility				0.906	.478
Q11					0.708	.618
Q23					0.479	.791
Q6	Segregation				3.460	.005*
Sixth/Medicine		45	2.73	1.91		
First/Pharmaceutical Sciences		50	3.88	1.83		
First/Veterinary Medicine		16	2.38	2.00		
Q15^a^					7.792	<.001
First/Medicine		33	3.15	1.82		
Sixth/Medicine		45	1.87	1.12		
First/Pharmaceutical Sciences		50	3.82	1.99		
Fifth/Pharmaceutical Sciences		32	3.06	1.56		
Q17^a^					6.680	<.001
Sixth/Medicine		45	1.36	0.65		
First/Pharmaceutical Sciences		50	2.9	1.85		

*Note. SD* = standard deviation.

a Multiple comparison performed by the Games-Howell test.

* *p* < .050.
